# Inducing an LCST in hydrophilic polysaccharides via engineered macromolecular hydrophobicity

**DOI:** 10.1038/s41598-023-41947-z

**Published:** 2023-09-09

**Authors:** Saniya Yesmin Bubli, Matthew Smolag, Ellen Blackwell, Yung-Chun Lin, John G. Tsavalas, Linqing Li

**Affiliations:** 1 Department of Chemical Engineering and Bioengineering, University of New Hampshire, Durham, NH 03824 USA; 2 Department of Chemistry, University of New Hampshire, Durham, NH 03824 USA; 3 Materials Science Program, University of New Hampshire, Durham, NH 03824 USA

**Keywords:** Phase transitions and critical phenomena, Biomaterials, Biomedical engineering, Chemical engineering

## Abstract

Thermoresponsive polysaccharide-based materials with tunable transition temperatures regulating phase-separated microdomains offer substantial opportunities in tissue engineering and biomedical applications. To develop novel synthetic thermoresponsive polysaccharides, we employed versatile chemical routes to attach hydrophobic adducts to the backbone of hydrophilic dextran and gradually increased the hydrophobicity of the dextran chains to engineer phase separation. Conjugating methacrylate moieties to the dextran backbone yielded a continuous increase in macromolecular hydrophobicity that induced a reversible phase transition whose lower critical solution temperature can be modulated via variations in polysaccharide concentration, molecular weight, degree of methacrylation, ionic strength, surfactant, urea and Hofmeister salts. The phase separation is driven by increased hydrophobic interactions of methacrylate residues, where the addition of surfactant and urea disassociates hydrophobic interactions and eliminates phase transition. Morphological characterization of phase-separated dextran solutions via scanning electron and flow imaging microscopy revealed the formation of microdomains upon phase transition. These novel thermoresponsive dextrans exhibited promising cytocompatibility in cell culture where the phase transition exerted negligible effects on the attachment, spreading and proliferation of human dermal fibroblasts. Leveraging the conjugated methacrylate groups, we employed photo-initiated radical polymerization to generate phase-separated hydrogels with distinct microdomains. Our bottom-up approach to engineering macromolecular hydrophobicity of conventional hydrophilic, non-phase separating dextrans to induce robust phase transition and generate thermoresponsive phase-separated biomaterials will find applications in mechanobiology, tissue repair and regenerative medicine.

## Introduction

Thermoresponsive materials derived from natural or synthetic macromolecules that undergo reversible phase transitions have attracted tremendous attention for a variety of biomedical applications including biomanufacturing additives^1^, drug delivery^2–6^, biosensors^7^, separation and purification process^8,9^, and tissue engineering^10–12^. Phase separation is a thermodynamically and kinetically driven process where a single homogeneous solution separates into two distinct phases via either a lower critical solution temperature (LCST) or an upper critical solution temperature (UCST), respective critical temperature points above and below which polymers undergo transition from solution to aggregates^13–15^. This phase transition behavior of thermoresponsive macromolecules can be controlled by a variety of factors that include the degree of polymerization^16^, polymer topologies^17^, hydrophilic to hydrophobic ratios^18^, and other generic stimuli such as temperature^19–21^, pH^22,23^, concentration^24^, molecular weight^25^, ionic strength and specific salt types^26,27^. Among these parameters, temperature is one of the most easily applied factors that readily modulates the solution properties of materials and as a means to provide a benchmark to engineer bio-responsive materials for various biomedical applications^28^.

Phase separation is known to be involved in many biological processes such as the formation of membraneless compartments in cells such as cytoplasm and nucleoplasm^29–32^. It has also recently been suggested that abnormal phase separation triggers protein aggregation, which plays a critical role in the context of neurodegenerative disease pathogenesis such as tau in Alzheimer disease (AD), alpha-synuclein in Parkinson, and RNA processing FUS protein in cancer development^29,30,33,34^. Multiple classes of thermoresponsive materials have been extensively synthesized and investigated to understand the fundamental mechanisms of molecular interactions that govern the aggregation process especially in synthetic polymers and recombinant proteins^35,36^. For example, poly(N-isopropylacrylamide) (pNIPAM) undergoes a reversible and rapid, coil-to-globule transition at a lower critical solution temperature of 32 °C in aqueous condition, making it a suitable candidate for controlled drug release^15,16,36^. Polyethylene glycol (PEG) exhibits a thermoresponsive behavior with a lower critical solution temperature approximately of 95 °C, a much higher transition temperature required for destabilization of hydrogen bonding and disruption of the surrounding hydration layer to trigger phase transitions due to its intrinsic hydrophilic characteristics^37^. Poly(oligo(ethylene glycol) methyl ether methacrylate) (POEGMA) is another major class of thermoresponsive polymer whose LCST can be varied by molecular structure in particular the length of the ethylene glycol side chains^38,39^. Although increasing molecular weight generally decreases the transition temperature^37^, it remains difficult to engineer transition temperatures in the physiological range due to limited chemical reactive groups. Recombinant polypeptides such as elastin-like polypeptides (ELPs) and resilin-like polypeptides (RLPs) are another category of thermoresponsive materials with reversible phase transitions mediated via a secondary conformational change from random coiled morphology to stabilizing β-sheet/β-turn structures upon temperature increase^35,40,41^. Recombinant strategies offer precise control of amino acid sequence, guest residue, molecular weight, and hydrophilic-hydrophobic ratio to achieve tunable transition temperatures^35,40–43^, however, the timeline from plasmid construction to protein expression with limited protein yield after purification creates additional challenges.

Recently, there has been an increasing interest in expanding natural thermoresponsive polysaccharides and developing simplified strategy to design novel thermoresponsive polysaccharide-based materials with precisely controlled phase transition. Polysaccharides effectively combine the chemical versatility and material processing efficiency from synthetic polymers with improved bioactivity and biocompatibility from natural biopolymers, offering great opportunities to engineer material properties. Accessing different molecular weights with a large number of available reactive moieties along the polysaccharide backbone permits systematic tuning and precise chemical modification to achieve desired material composition and structure/property relationships. Substantial effort has been focused on developing polysaccharide-based thermoresponsive block copolymers such as dextran-PCL-HEMA, alginate-g-PNIPAM, chitosan-pluronic F127 and others, through chemical conjugation of synthetic thermoresponsive polymers to natural non-thermoresponsive polysaccharides^1^. Although the thermal behaviors of these block copolymers can be manipulated, it remains challenging to systematically and precisely modulate their transition temperatures due to the top-down approach utilizing polysaccharide as building blocks for the generation of thermoresponsive block copolymers. Therefore, these difficulties necessitate the development of more straightforward synthetic ways to develop new thermoresponsive polysaccharides with controlled phase separation and transition temperatures.

To convert non-thermoresponsive polysaccharides into thermoresponsive materials without the requirement of conjugating polymers to generate block-copolymers, simplified synthetic approaches will be advantageous. In this work, we exploit the use of one step chemical modification to introduce hydrophobic residues to the hydrophilic backbone of dextran, a simple but controlled method, to increase the overall macromolecular hydrophobicity that triggers effective phase separation. Dextran is a non-toxic, biocompatible, biodegradable and FDA approved macromolecule and thus an attractive candidate for tissue engineering applications^1,13,44–46^. By conjugating methacrylates to the hydroxyl groups of the dextran backbone, a gradual increase in hydrophobicity of modified dextran macromers results in induced phase transitions of previously hydrophilic, non-phase separating dextran macromolecules. The lower critical solution temperature and phase-separated microdomain sizes can be further tuned by varying material compositions and solution conditions, as characterized via dynamic light scattering (DLS) and UV–Vis spectroscopy. The morphology and stability of the microdomains were also confirmed by scanning electron microscopy (SEM) and flow imaging microscopy (FlowCam). Cultures of human dermal fibroblasts suggested well tolerated cytocompatibility of phase separated domains. The thermoresponsive Dex-MA macromers permitted UV-initiated crosslinking to form heterogeneous and microstructured hydrogels by capturing the microdomains upon phase separation. Unlike conventional polymer-grafting to polysaccharide copolymer, our bottom-up approach provides a unique way to establish a class of novel and finely-tuned thermoresponsive polysaccharides and offers a simplified strategy to capture phase separation and microdomains in hydrogels that have potential in mechanobiology and wound healing applications.

## Methods

### Materials

Dextran (from *Leuconostoc mesenteroides*, Mw: 40–500 kDa) was purchased from MP Biomedicals (Irvine, CA). Dimethyl sulfoxide (DMSO, anhydrous), glycidyl methacrylate (GMA), 4-(N,N-dimethylamino) pyridine (DMAP) and all other reagents were purchased from Sigma Aldrich (St. Louis, MO) and used as received, unless otherwise stated. Dialysis tubing semi-permeable membrane with a molecular weight cut off 10,000 Da was purchased from Thermo Fisher Scientific (Waltham, MA).

### Chemical synthesis of methacrylated dextran (Dex-MA)

To synthesize methacrylated dextran with various degrees of functionalities, dextran (2.0 g, Mw: 40–500 kDa) was dissolved in 10 mL of anhydrous dimethyl sulfoxide (DMSO) with the addition to 0.2 g of base catalyst 4-dimethylamino pyridine (DMAP) and the required molar equivalent of glycidyl methacrylate (GMA, density = 1.042 g/mL at 25 °C). The mixture solution was kept constant at 45 °C and stirred for 24 h. After stirring, the reaction solution was pipetted dropwise into a 200 mL of ice-chilled isopropanol to precipitate modified dextran. The precipitation was then collected via centrifugation and subsequently re-dissolved and dialyzed against 4 L of milli-Q water at a temperature of 4 °C preceding lyophilization. Purified methacrylated-dextran (Dex-MA) was stored at − 20 °C until use. The functionality and degree of methacrylation of Dex-MA were analyzed using nuclear magnetic resonance spectroscopy (NMR, a 700 MHz Bruker BioSpin spectrometer, in D_2_O/DMSO), where the degree of methacrylation confirmed to achieve 40–88% modification depending on the initial molar equivalent of glycidyl methacrylate to glucopyranose. The peaks at 5.75 and 6.2 ppm represent the protons at the double bond of the GMA group.

### Fourier transform infrared (FTIR) spectroscopy

FTIR spectroscopy experiments were performed using a Thermo Nicolet iS10. Spectra taken at a resolution of 4 cm^−1^ from 400 to 4000 cm^−1^ were obtained by signal averaging 32 scans. Dried samples of non-modified dextran and Dex-MA were loaded and pressed to get a good contact with the diamond crystal plate for collecting the spectra. For data collection and analysis, Omnic software was used. FTIR transmissions were recorded at 1706 cm^−1^ for carbonyl groups, and double bonds of GMA were detected at 813 cm^−1^ and 1640 cm^−1^.

### Dynamic light scattering (DLS) characterization

The average size change of dextran-based particles as a function of temperature was analyzed via dynamic light scattering (DLS) using a Malvern Zetasizer Nano ZS instrument (Malvern Instruments Ltd, Worcestershire, UK). Dextran samples (1 mL) at various polysaccharide concentrations (0.1–10 mg/mL) were prepared in phosphate-buffered saline solution (PBS, pH ~ 7.4) in disposable or glass cuvettes before measurement. The average hydrodynamic diameter was measured at different material properties and solution conditions (e.g., degree of methacrylation, dextran molecular weight, polysaccharide concentration, type of Hofmeister salts, surfactant and urea) over a range of temperature with a 2 °C/min interval heating scanning rate. The transition temperature was determined as the temperature at which a sharp increase in particle size is observed. The accuracy of the transition temperature is approximately ± 1 °C.

### Turbidity characterization via UV–Vis spectroscopy

The turbidity of dextran samples with various material properties and solution conditions was obtained by measuring solution absorbance at 400 nm wavelength as a function of temperature. All samples were characterized in a glass cuvette for turbidity assay using a Cary 3500 UV–Vis spectrophotometer (Agilent Technologies, Santa Clara, CA) equipped with a temperature-controlled cell holder. The solution temperature was increased from 10 to 60 °C at a constant 1 or 2 °C/min interval heating scanning rate until a plateau was achieved in absorbance value. A baseline absorbance curve of PBS solution was collected as a reference before each sample run. The transition temperature was determined considering the start of the rise in the absorbance value of the UV–Vis spectra.

### Scanning electron microscopy (SEM)

Images of phase separated microdomains were collected on a SEM microscope (Tescan Lyra 3 GMU, Warrendale, Pennsylvania). Dextran samples (30 µL each) at 1 mg/mL polysaccharide concentrations were prepared in phosphate-buffered saline solution (PBS, pH ~ 7.4) and heated at various temperatures 24 °C, 45 °C and 60 °C. For analysis in SEM, aluminum stub specimen holders were prepared with carbon conductive paint coating and covered with cover slips. Dex-MA solutions were deposited onto the coverslip in dropwise manner and incubated for 12–24 h to prepare dry films. The dried samples were then sputter-coated with 15 nm gold palladium prior imaging. The SEM experiments were performed at 3 kV accelerating voltage and the images were captured using a secondary electron detector in the sample chamber. The average size of phase-separated particles were quantified via ImageJ (NIH software, Bethesda, MD).

### Flow imaging microscopy (FlowCam 8100)

Particle morphology, counts and size were captured in FlowCam 8100 (Yokogawa Fluid Imaging Technologies, Scarborough, Maine) imaging and data analysis was performed using Visual Spreadsheet software. The 88% Dex-MA samples were prepared in PBS at three different concentrations: 10, 1 and 0.1 mg/mL. The solution temperatures were 24 °C, 45 °C and 60 °C for 10, 1 and 0.1 mg/mL concentrations, respectively. After that 1 mL aliquots of each sample were inserted into the flow cell at approximately 150 µL/min flow rate. The flow cell directs the sample containing the particles past the microscope optics by capturing thousands of images of particles per second. The instrument was equipped with a 10 × objective and grayscale camera to capture the particles in the solution stream. The Visual Spreadsheet software automatically separates the particle images from the background as soon as each frame of the camera's field of view is collected. The frequency of microdomain counts is also directly proportional to concentration; for instance, it was found that 0.1 mg/mL concentration had around 32,000 counts of domains while 10 mg/mL concentration had approximately 115,000 counts based on the particle size distribution.

### In vitro cell culture

Human dermal fibroblasts (HDFs, passage ~ 7–9, Lonza, Basel, Switzerland) were cultured in fully supplemented Dulbecco's Modified Eagle's Medium (DMEM) (Lonza, Basel, Switzerland). All cells were cultured in a humidified incubator at 37 °C with 5% CO_2_. HDFs were cultured to 80% confluency before seeding in 35 mm MatTek glass bottom dishes (300,000 cells per dish). After culturing for 24 h, cell media was replaced with 2 mL DMEM media containing 88% Dex-MA at 10 mg/mL concentration for an additional 24 h before fixation.

### Live/dead assay

Human dermal fibroblasts (HDFs, passage 4–8) were cultured in DMEM media to 80% confluency on TCPS before seeding in 35 mm MatTek glass bottom dishes (100,000 cells per dish). After culturing for 24 h, cell media was replaced with 2 mL DMEM media containing 10 mg/mL 88% Dex-MA and live/dead cytotoxicity assay was performed after culturing HDFs in Dex-MA containing DMEM media for additional 24 h. Calcein AM (2 μM) plus Ethidium homodimer-1 (20 μM) were mixed in 10 mL PBS (pH = 7.4) to prepare the live/dead staining solution. Cultured cells were incubated with live/dead solution for 10–15 min at room temperature prior to imaging. Fluorescent images were acquired using a Nikon A1R HD confocal microscope. Unless otherwise specified, images were processed and presented as maximum intensity projections.

### Fluorescent staining and confocal microscopy

Human dermal fibroblasts (HDFs) cultured with Dex-MA solution were fixed with 4% paraformaldehyde (PFA) in PBS at room temperature for 15 min. To visualize cell morphology and the organization of the actin cytoskeleton, HDFs were stained with phalloidin-Alexa Fluor 647, 1:1000 (Life Technologies, Carlsbad, CA) and counterstained for nuclei with Hoechst (1:500) overnight at 4 °C. Fluorescent images were acquired using a Nikon A1R HD confocal microscope (Nikon Instruments Inc., Melville, New York). Unless otherwise specified, images are processed and presented as maximum intensity projections.

### Cell proliferation assay

Fixed human dermal fibroblasts (HDFs) cultured with and without Dex-MA solution were permeabilized with 0.1% triton X-100 in PBS at room temperature for 20 min, blocked with 5wt% goat serum in 0.01% triton X-100 at 4 °C overnight, and incubated with primary antibody Ki67 mouse monoclonal antibody (1:500, Abcam ab15580) in blocking buffer for 24 h at 4 °C. After 24 h, HDFs were incubated with goat anti-mouse Alexa Fluor 488 (1:1000, Life Technologies); counterstained for nuclei with Hoechst (1:500) and for actin cytoskeleton with phalloidin-Alexa Fluor 647 (1:1000, Thermo Fisher Scientific, Waltham, MA) in blocking buffer. Fluorescent images were acquired using a Nikon A1R HD confocal microscope. Unless otherwise specified, images are processed and presented as maximum intensity projections using Image J.

### Hydrogel formation

Lyophilized Dex-MA (Mw: 86 kDa, f = 88%) were dissolved in PBS (pH = 7.4) solutions at 50 mg/mL concentrations. A 10 mg/mL stock solution of the photo-initiator 2-Hydroxy-4′-(2-hydroxyethoxy)-2-methylpropiophenone (Irgacure 2959, Sigma-Aldrich, St. Louis, MO) was prepared in ethanol. 2 µL of photo-initiator solution was added to 100 µL of phase-separated cloudy Dex-MA solution, and the mixture was pipetted gently to ensure proper mixing. The resulting solution was transferred to a MatTek dish and photo-crosslinked using an Omnicure S2000 UV lamp (EXFO) with 365 nm wavelength at 25 mW/cm^2^ intensity for 1 min under argon to create UV-initiated hydrogels. To make non-phase separated hydrogels, a separate hydrogel precursor solution was prepared and cooled to 4 °C to eliminate the phase separation of Dex-MA solution to generate homogeneous, non-phase separated hydrogels.

### Statistical analysis

Statistical analysis was performed using Prism-GraphPad, where multigroup analysis was determined by a one-way analysis of variance (ANOVA) followed by Tukey-HSD post-hoc test on all data set. Dual group analysis was performed using an unpaired Student’s *t*-test. Statistical significance is indicated by **** which corresponds to P values < 0.0001.

## Results

### Conjugation of hydrophobic adducts induces an LCST in Dex-MA

**Figure 1:**
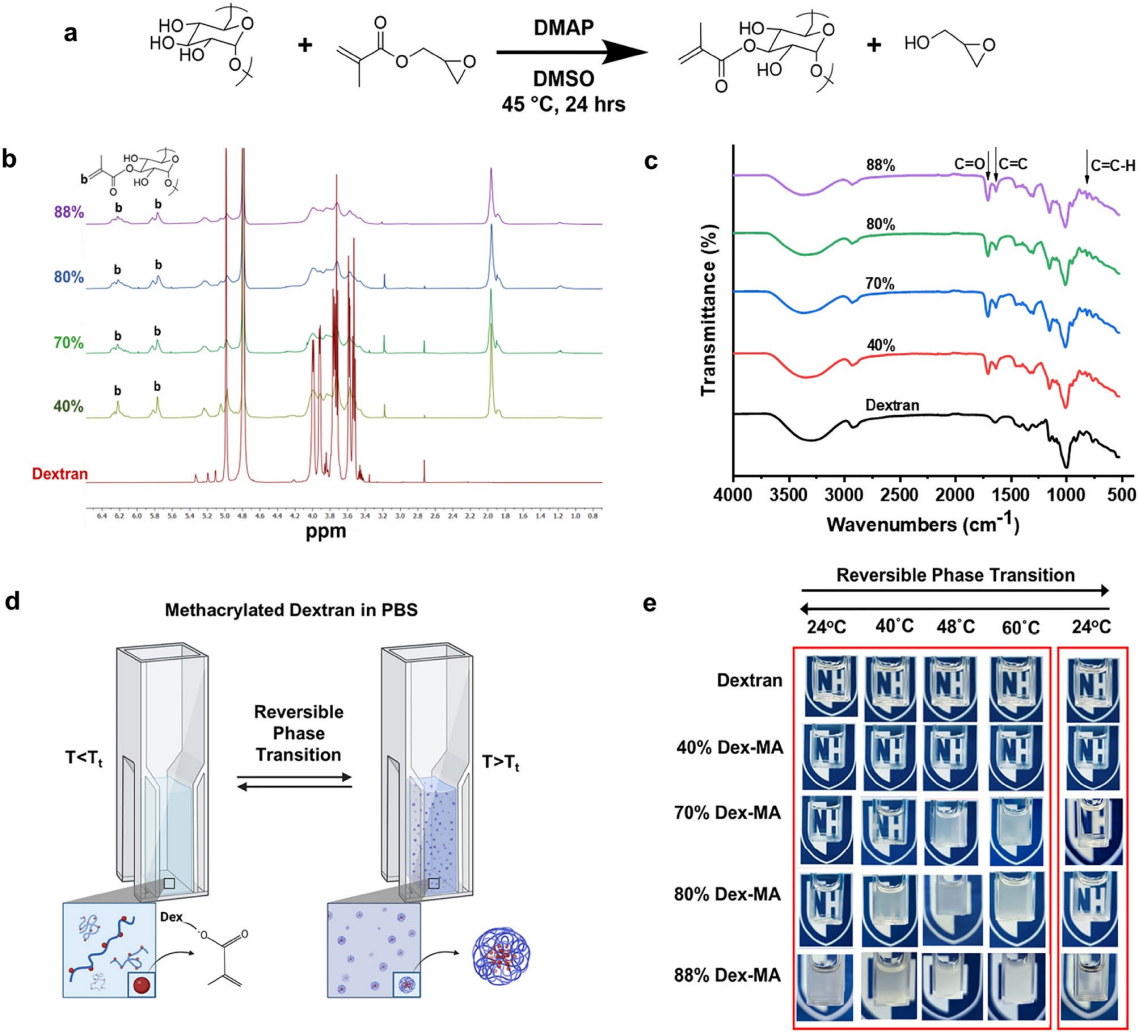
The degree of methacrylate functionality regulates reversible phase separation of Dex-MA in aqueous solutions. (**a**) Schematic of chemical synthesis of dextran methacrylate. (**b**) H-NMR spectrum of dextran and Dex-MA (Mw: 86 kDa) with different methacrylation percentage in D_2_O. (**c**) FTIR spectrum of dextran and Dex-MA (Mw: 86 kDa) with different degree of methacrylation. (**d**) Schematic of temperature triggered reversible phase transition of Dex-MA solutions. (**e**) Images of various Dex-MA in PBS (pH ~ 7.4) solutions (~ 86 kDa, 10 mg/mL) as a function of degree of methacrylation incubated at various temperatures to induce phase transition.

To engineer a phase transition in hydrophilic polysaccharides, we hypothesize that increasing the hydrophobicity of the polymer backbone will trigger a reversible phase separation that exhibits a lower critical solution temperature (LCST). Dextran, a neutral and hydrophilic homo-polysaccharide, was selected as the base material and chemically modified with glycidyl methacrylates (GMA) via reactive hydroxyl groups to yield methacrylated dextran (Dex-MA, Fig. 1a). The synthesis of dextran methacrylate incorporates a transesterification reaction between glycidyl methacrylate (GMA) and dextran, which results in the direct attachment of the methacryloyl moiety from GMA to dextran through an ester linkage^47^. In addition, the instability of glycidyl methacrylate's epoxy group renders it susceptible to nucleophilic attack by hydroxyl groups in the presence of catalysts^48^. The degree of methacrylation can be easily tuned via changing the molar ratio of GMA to glucopyranose residues in order to achieve a wide range of functionality (40–80%), confirmed by NMR and FTIR (Fig. 1b,c and Fig. S1) To explore whether changing the overall hydrophobicity of modified dextran can induce a phase transition, solutions of Dex-MA at 10 mg/mL concentration with different methacrylation degrees (0–88%) were heated and the phase transition behavior was monitored over time (Fig. 1d,e). Methacrylated dextrans with different functionalities (f = 40%, 70%, 80% and 88%; Mw ~ 86 kDa) were compared to non-modified dextran. Lowering the solubility of Dex-MA by increasing the solution temperature resulted in an obvious phase separation that generated two immiscible liquid phases, a dense Dex-MA phase, and a dilute equilibrium phase, therefore the cloudy appearance of the Dex-MA solutions denotes the phase transition. Non-modified dextran and 40% modified Dex-MA samples showed no phase separation across the range of temperatures tested, depicted by the clear visibility of the UNH logo (Fig. 1e). Increasing temperature of both the 70% and 80% modified Dex-MA solutions induced obvious phase separation followed by rapid clearance of cloudiness (~ mins) upon returning to room temperature, indicative of a reversible phase transition. Interestingly, 88% modified Dex-MA solutions displayed a dense cloudy appearance and maintained phase separation at room temperature, showing that its LCST is below room temperature. The correlation between the degree of methacrylation and associated phase transition suggests that increased methacrylate content on hydrophilic dextran backbone converts overall macromolecular hydrophobicity that triggers reversible phase transitions.

### Tunable solution parameters modulate the LCST

**Figure 2:**
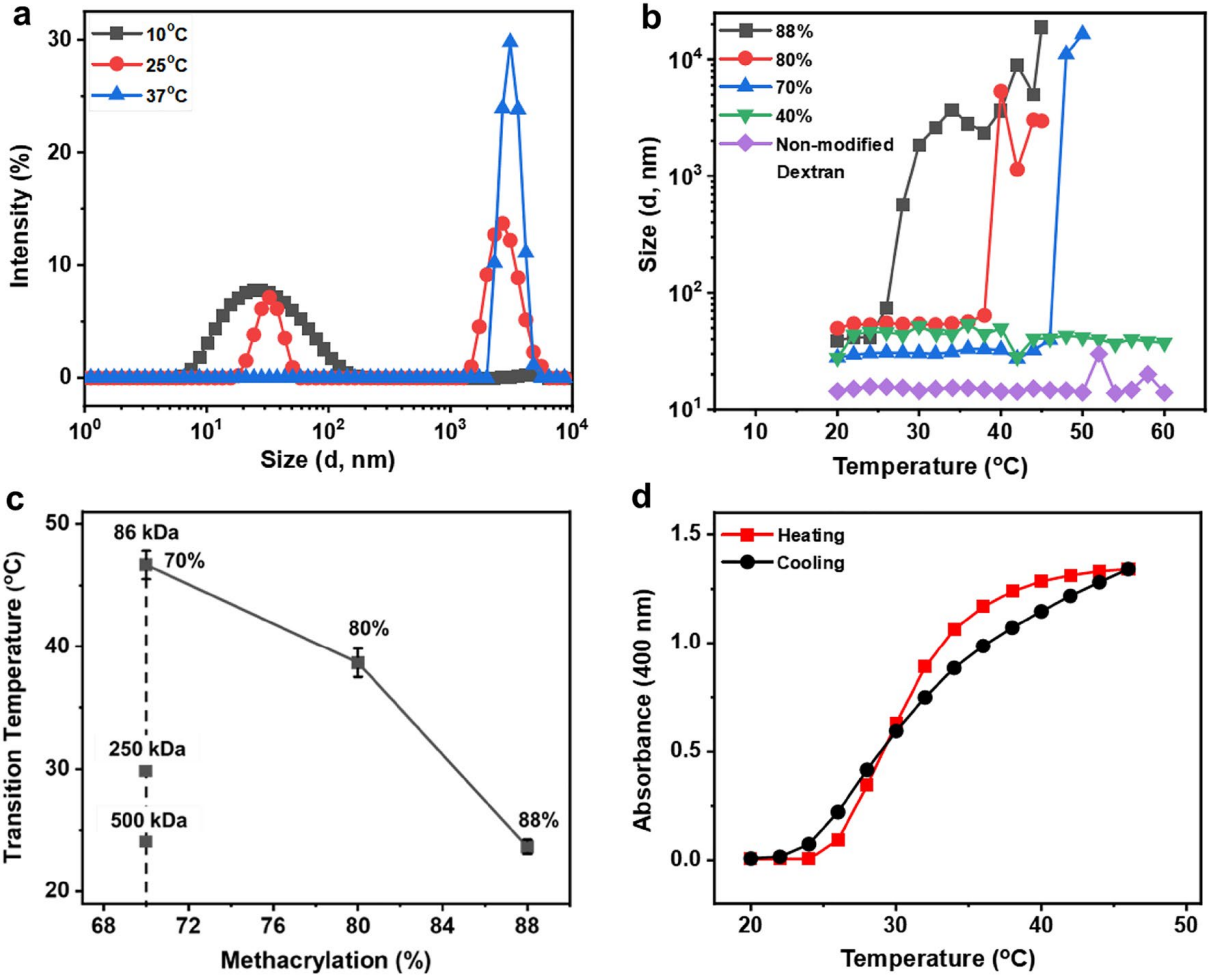
Dynamic light scattering and UV–Vis spectroscopy characterization of Dex-MA phase transition in aqueous solutions. (**a**) Intensity (%) of Dex-MA in PBS (pH ~ 7.4) solutions as a function of size at different temperatures (Mw: 86 kDa, f = 88%). (**b**) Tunable transition temperatures via controlling degree of methacrylation in PBS (pH ~ 7.4) at 10 mg/mL solution concentration (Mw: 86 kDa). (**c**) Molecular weight (86 kDa, 250 kDa, 500 kDa) and the degree of methacrylation dependent LCST of dextran (86 kDa) in PBS (pH ~ 7.4) at 10 mg/mL sample concentration. (**d**) Reversible heating and cooling profile of Dex-MA with respect to absorbance, in PBS (pH ~ 7.4) at 1 mg/mL sample concentration (Mw: 86 kDa, f = 88%).

To quantitatively characterize and investigate parameters that modulate the reversible phase transition of Dex-MA in aqueous solutions, dynamic light scattering (DLS) and temperature-controlled UV–Vis spectrophotometer were employed to monitor transition temperatures and the change of microdomains size over multiple solution conditions. The thermoresponsive behavior of 88% modified Dex-MA (Mw ~ 86 kDa) at 10 mg/mL concentration in PBS was confirmed via DLS by tracking the changes of particle size over a range of temperatures. Increasing solution temperature from 10 °C to 25 °C to 37 °C resulted in a significant increase in particle size shifting from roughly 30 nm at 10 °C, to ~ 2600 nm at 25 °C, and to over 3000 nm at 37 °C, indicative of the formation of microdomains upon phase transition (Fig. 2a). We acknowledge that DLS-based scattering characterization method is restricted by the wavelength and the concentration of the solution, and not always accurately presents diameters in the range above 1000 nm, however, we simply employed the changes in diameters as an indicative of the onset of phase transition. To examine the effect of the degree of methacrylation on phase separation and identify the transition temperatures, Dex-MA solutions with a varying degree of methacrylation were characterized via DLS over a range of temperatures. Non-modified dextran and 40% Dex-MA showed a constant particle size (< 30 nm) over the range of temperature tested; where 70%, 80% and 88% Dex-MA solutions exhibited a sharp increase in particle size at the onset of phase separation with transition temperatures of 47 °C, 41 °C and 24 °C respectively (Fig. 2b,c). These transition temperatures are consistent with the visual cloudy point observed in Fig. 1e. To explore the impact of molecular weight on transition temperature, we maintained the degree of methacrylate constant (f = 70%) and characterized transition temperatures of Dex-MA solutions at 250 kDa and 500 kDa. It was observed that increasing molecular weight decreases transition temperatures, with 250 kDa and 500 kDa Dex-MA exhibiting lower transition temperatures around 30 °C and 24 °C respectively compared to 86 kDa Dex-MA solutions (Fig. 2c). To validate the reversibility of phase transition behavior exhibited by Dex-MA materials, heating and cooling cycles of Dex-MA solutions were performed. Temperature-controlled UV–Vis spectroscopy was employed to conduct turbidity assays via monitoring the absorbance of Dex-MA solutions at 400 nm as a function of temperature with 1 °C/min heating and cooling rate. Dex-MA solutions were heated above the known transition temperature to trigger the phase separation with an increase in UV absorbance, followed by the disappearance of cloudiness with a decrease in absorbance upon cooling. The overlap in UV absorbance between heating and cooling cycle without significant hysteresis suggests an efficient LCST behavior where reversible transition of phase separation is distinct from typical temperature-induced non-reversible aggregation (Fig. 2d).

**Figure 3:**
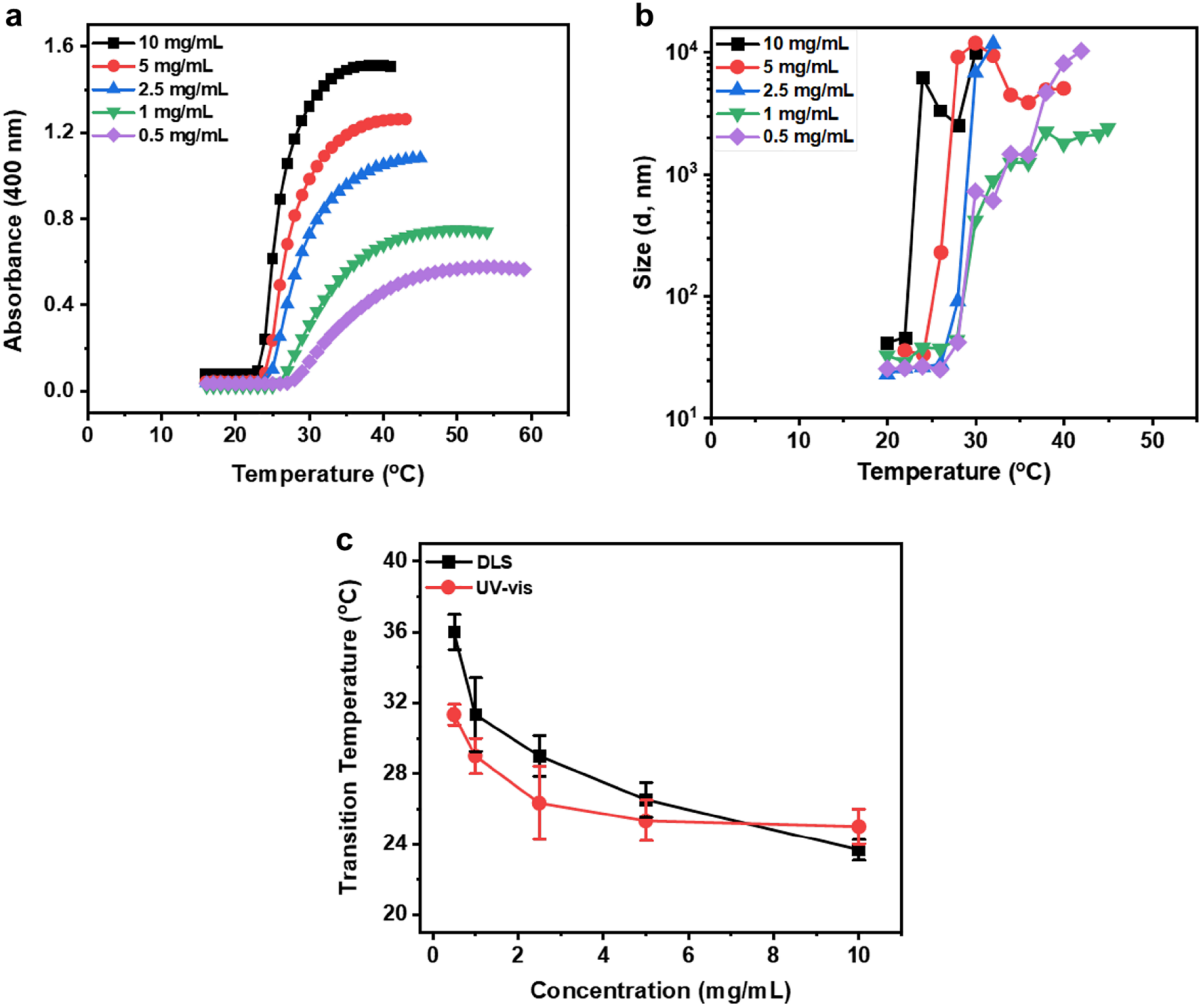
Polysaccharide solution concentration regulates phase transition. (**a**) Characterization of phase transition temperatures as a function of Dex-MA solution concentrations (Mw: 86 kDa, f = 88%) in PBS (pH ~ 7.4) via monitoring absorbance at 400 nm in UV–Vis and (**b**) particle size in DLS. (**c**) Summary of transition temperatures at various Dex-MA solution concentrations in PBS (pH ~ 7.4) quantified from both DLS and UV–Vis experiments. Error bars represent the standard deviation of three replicates.

To explore the impact of Dex-MA concentration on the phase transition, we employed both temperature-controlled UV–Vis and DLS to characterize the phase transition and compared the transition temperatures over a range of Dex-MA concentrations (0.5–10 mg/mL). Increasing temperature induces phase separation across all concentrations tested, confirmed by the drastic increase in the UV absorbance at 400 nm (Fig. 3a) and the particle size via DLS (Fig. 3b). The transition temperatures for solutions with various Dex-MA concentrations are summarized in Fig. 3c, with an increase of polysaccharide concentration correlating with a lower transition temperature. This characteristic concentration dependence of transition temperature is consistent with both methods with a slight variation at lower Dex-MA concentrations. The slight differences of the transition temperatures at the same Dex-MA solution concentrations between UV–Vis and DLS are likely due to the methods used for calculation, where in UV–Vis the transition temperature is determined as the inflection in the absorbance spectrum and in DLS it is taken at the point where the particle size shows a sharp increase. While these are the most common methods to characterize this phase transition, we note that these scattering based techniques are observing the aggregation of the phase separated hydrophobic morphology of the systems and not specifically the initial expulsion of water and intramolecular collapse of the chains (which would be observed as a decrease in scattering size). We are currently studying alternative characterizations not based in scattering to also indicate this onset of the phase separation (the initial chain collapse), yet the methods reported here are indeed consistent with the traditional and common analyses.

### Addition of surfactant and urea increase the LCST of Dex-MA solutions

**Figure 4:**
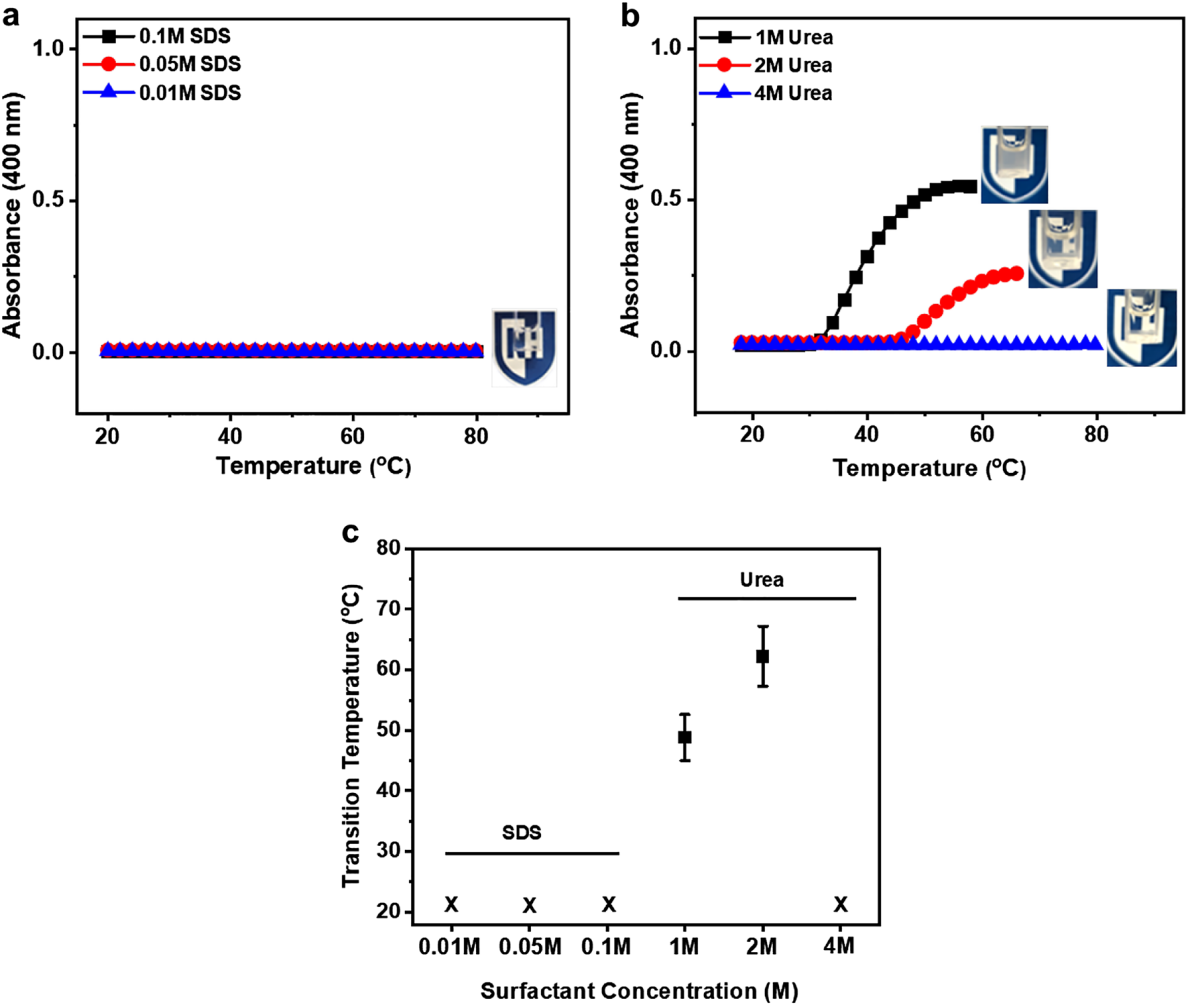
Effects of SDS and urea on phase transition of Dex-MA. (**a**) Turbidity assay of Dex-MA (Mw: 86 kDa, f = 88%) in 0.1 M, 0,05 M and 0.01 M SDS at 1 mg/mL sample concentration via UV–Vis. (**b**) Turbidity profile of Dex-MA (Mw: 86 kDa, f = 88%) in 1 M, 2 M and 4 M urea at 1 mg/mL sample concentration via UV–Vis. (**c**) Comparison of SDS and urea effects on transition temperature. X refers to no transition temperature observed across the tested temperature range.

To validate whether hydrophobic-hydrophobic interaction is the driving force for the temperature-induced phase separation in the engineered Dex-MA system, we evaluated the effects of urea and sodium dodecyl sulfate (SDS) on the LCST of Dex-MA solutions. Turbidity assay of Dex-MA solutions at various concentrations of SDS and urea was conducted, and UV absorbance as a function of temperature was plotted (Fig. 4a,b) and their transition temperatures were summarized in Fig. 4c. The phase separation behavior of Dex-MA solutions is sensitive to the addition of both SDS and urea. Interestingly, the addition of SDS (Fig. 4a), although at much lower concentrations (0.01–0.1 M), completely eliminated the phase transition across all conditions tested even at temperatures up to 80 °C, confirming hydrophobic interactions introduced via chemically conjugated methacrylates as a major contributor for Dex-MA aggregation and microdomain formation. Increasing urea concentration from 1 to 2 M shifted the transition temperature from 49 to 62 °C, and further increasing urea concentration to 4 M resulted in a clear disappearance of the phase transition (Fig. 4b), suggesting a role of hydrogen bonding in the aggregation process^49,50^. This general observation was also confirmed via DLS, where Dex-MA solutions with an addition of 0.1 M SDS or 4 M urea yielded an average of hydrodynamic size below 50 nm and showed no sign of aggregation. In comparison, Dex-MA samples without any surfactant or at 1 M urea exhibited higher intensity peaks with a larger hydrodynamic size (> 200 nm), suggesting the occurrence of phase separation (Fig. S2). The addition of urea and SDS offer additional means to modulate the phase transition and reveals a potential mechanism that hydrophobic-hydrophobic association regulates macromolecular aggregation during phase separation.

### Cations and anions of Hofmeister salts differentially regulate phase transition

**Figure 5:**
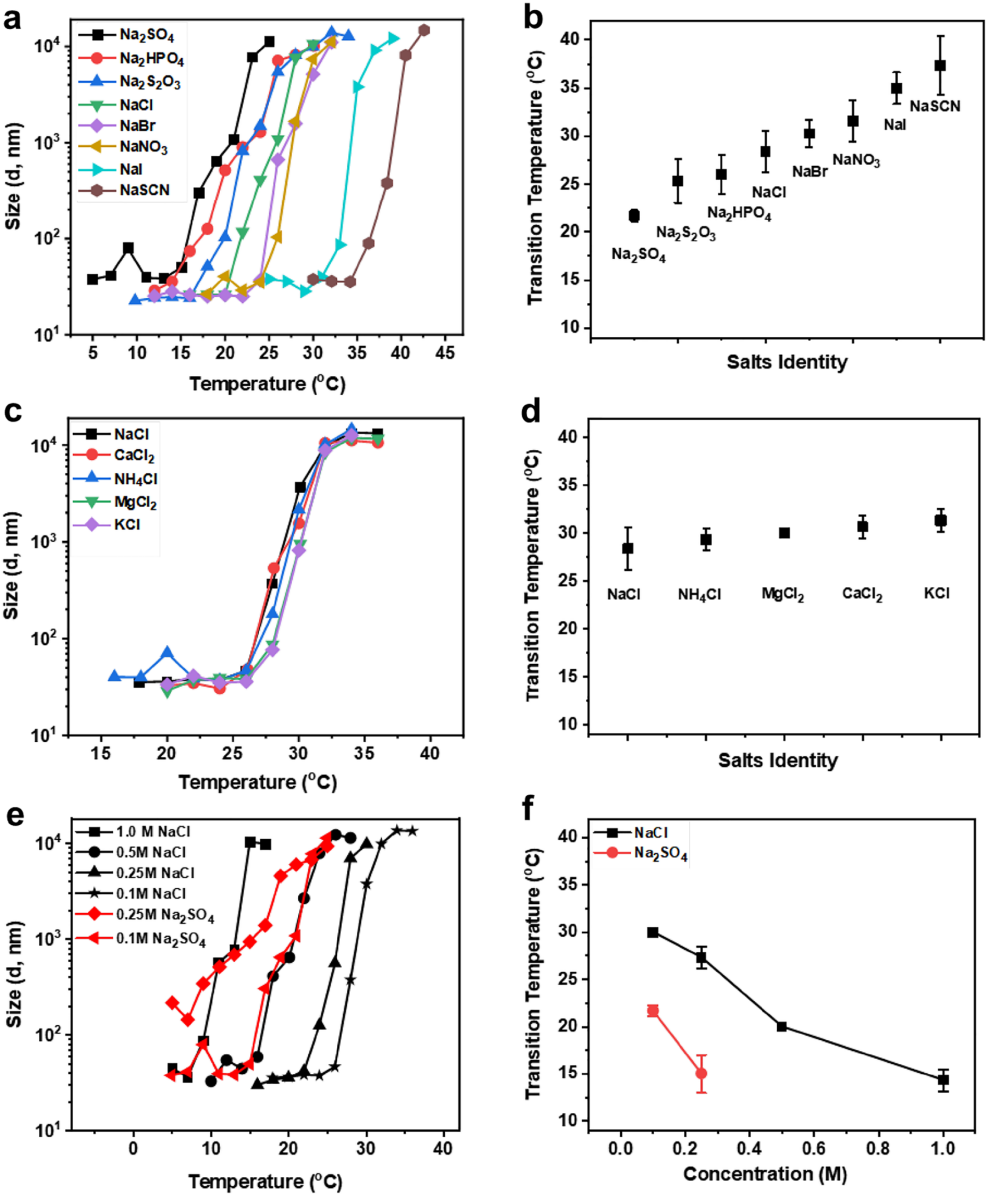
Hofmeister salts with different cations and anions differentially effect Dex-MA phase transition temperatures. (**a**) Hydrodynamic radius profiles of Dex-MA (Mw: 86 kDa, f = 88%) samples as a function of temperature under various salts, anions (0.1 M) conditions; (**b**) summary of the impact of Hofmeister salts with different anions; (**c**) Hydrodynamic radius profiles of Dex-MA (Mw: 86 kDa, f = 88%) samples as a function of temperature under various salts, cations (0.1 M) conditions (**d**) summary of the impact of Hofmeister salts with different cations and (**e**) Hydrodynamic radius profiles of Dex-MA (Mw: 86 kDa, f = 88%) samples with different concentrations of NaCl (0.1, 0.25, 0.5 and 1.0 M) and Na_2_SO_4_ (0.1 M and 0.25 M) salts solutions; (**f**) comparison of the impact of NaCl and Na_2_SO_4_ concentrations on the phase transition temperatures of Dex-MA solutions. Error bars represent the standard deviation of three different trials.

To investigate whether the aggregation process of the engineered Dex-MA system during phase separation is affected by other cosolutes, we analyzed the effects of a series of Hofmeister salts on the phase transition of Dex-MA solutions. Hofmeister classifies salt ions according to their ability to change the solubility of macromolecules^51,52^. The impact of cationic and anionic specificity on the thermoresponsive behavior of Dex-MA systems were evaluated in the presence of different salts chosen from the Hofmeister series at 0.1 M salt concentration. The hydrodynamic radius for different anions and cations was plotted as a function of temperature. In general, the phase separation behavior of Dex-MA solutions is more sensitive to the anions than the cations. Specifically, the addition of SO_4_^2^ to Dex-MA solutions resulted in a significant salting out effect and reduced transition temperatures from 32 °C (in PBS) to 21 °C, whereas the addition of SCN^−^ triggered an apparent salting in effect and increased transition temperature to 40 °C (Fig. 5a,b). This anions-dependent phase transition behavior of Dex-MA solutions is consistent with the expected behavior of ionic salts in the Hofmeister series, suggesting the role of anions to strongly interfere with the aggregations process. On the other hand, only slight variations in transition temperatures (26–32 °C) were observed across all cationic conditions measured (Fig. 5c,d), indicating a lower capability of cations to alter phase transition. To further characterize the impact of different ionic strengths on the transition temperature of Dex-MA solutions, NaCl and Na_2_SO_4_ at various ionic concentrations were tested and the transition temperatures are summarized in Fig. 5e,f. Increasing the NaCl salt concentration from 0.1, 0.25, 0.5 to 1 M yielded a continuous reduction in transition temperature from 30 to 14 °C respectively, as expected. Interestingly, adding Na_2_SO_4_ salts to Dex-MA solutions showed a similar but significant effect on reducing the LCST at lower concentrations with a transition temperature of 22 °C at 0.1 M and 15 °C at 0.25 M compared to those at the same NaCl concentrations, however, higher Na_2_SO_4_ concentration (both 0.5 and 1 M) caused Dex-MA to precipitate out of the solution, suggesting the stronger ionic strength of sulphate salts. The addition of different salts from Hofmeister series provides significant options to modulate the macromolecular aggregation process and impacts the phase transition temperatures of thermoresponsive polysaccharides.

### Phase separation mediates the formation of microdomains

**Figure 6:**
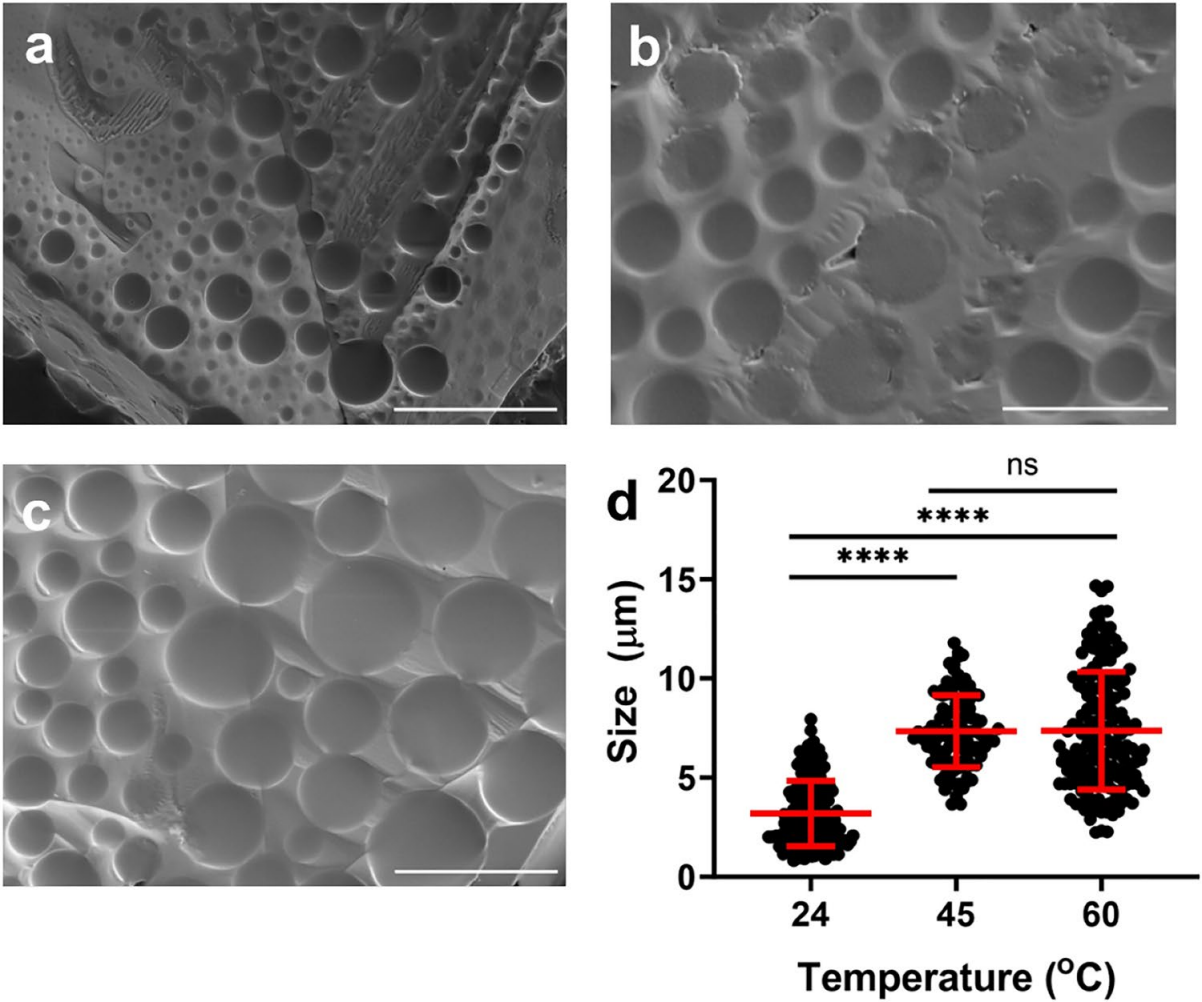
Characterization of phase separated microdomains in the dry Dex-MA samples via scanning electron microscopy. (**a**) Representative SEM images of Dex-MA (Mw: 86 kDa, f = 88%, 1 mg/mL in PBS (pH ~ 7.4)) samples dried at 24 °C below the transition temperature, (**b**) at 45 °C and (**c**) 60 °C above the transition temperature; (**d**) Microdomains size distribution at various temperatures with statistical analysis. Scale bars: 20 µm (****P < 0.0001).

To reveal the morphology and size of phase separated microdomains of Dex-MA samples, we performed scanning electron microscopy (SEM) and flow imaging microscopy experiments at temperatures below and above the phase transition. Representative SEM images were captured (Fig. 6a–c) at various temperatures and quantitative analysis of particle size distribution was summarized in Fig. 6d. Although well-defined microdomains were observed in all conditions, dry Dex-MA samples prepared at 24 °C (below the transition temperature) contained microdomains with sizes around 3 µm in diameter, which is significantly smaller compared to samples processed at 45 °C and 60 °C (above the transition temperature), which contained larger microdomains with an average diameter size of 9 µm. The microdomain size at ambient temperature resulted in significant difference with elevated temperatures (45 °C and 60 °C, P < 0.0001), however, no significant difference in size was observed within domains size at 45 °C and 60 °C, which is likely due to increased Dex-MA concentration reducing the LCST from solution drying during SEM sample preparation.

**Figure 7:**
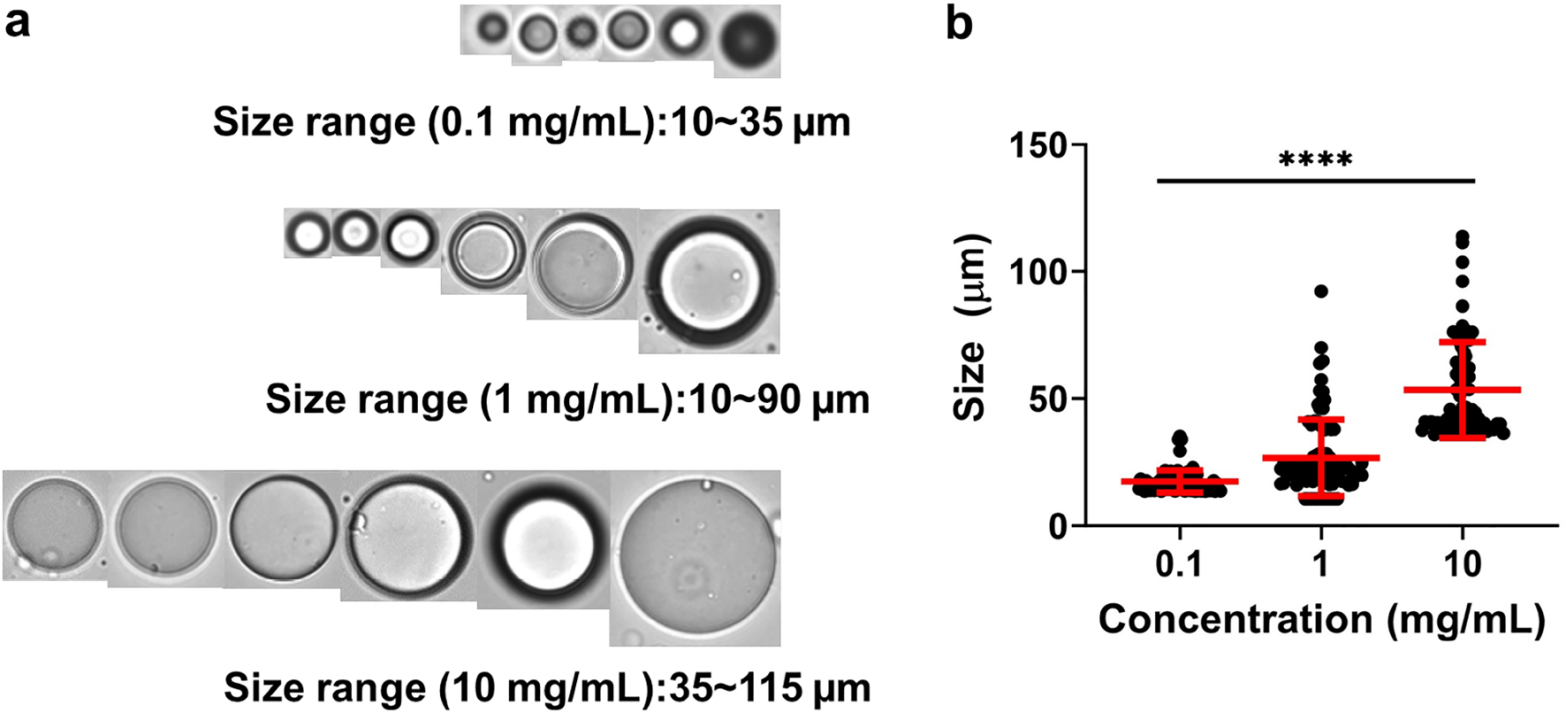
Characterization of phase separated microdomains in aqueous Dex-MA solution via flow imaging microscopy. (**a**) Representative FIM images of Dex-MA in PBS (pH ~ 7.4) solutions (Mw: 86 kDa, f = 88%) at 0.1 mg/mL, 1 mg/mL and 10 mg/mL concentration. (**b**) Particles size distribution at various Dex-MA concentrations with statistical analysis (****P < 0.0001).

To better understand and characterize the phase separation and microdomains formation in Dex-MA solutions, we employed a solution-based FlowCam imaging microscopy (FIM) to capture the high-resolution images to analyze the shape, size and counts of subvisible microdomains during the real-time phase separation. FlowCam captures large quantities of particles using the combination of microfluidics and light microscopy. Visual Spreadsheet is a particle analysis software and was used to analyze the results and images. Three different concentrations of Dex-MA (86 kDa, f = 88%) samples in PBS were measured in FlowCam, and representative microdomain images were captured and plotted in Fig. 7. It was observed that uniform microdomains were present across all three Dex-MA concentrations (Fig. 7a, Fig. S3), with a direct correlation that increasing Dex-MA concentration from 0.1, to 1 and to 10 mg/mL increases domains size, ranging from ~ 17 to 27 µm and to approximately 53 µm, respectively (Fig. 7b). The size information is consistent with a typical concentration dependent behavior and can be used as another parameter to modulate domain size upon phase transition. The microdomains in the solution stream at different concentrations exhibited significant differences in size (P < 0.0001).

### Cytotoxicity analysis to evaluate the effects of phase separated Dex-MA on cells viability

**Figure 8:**
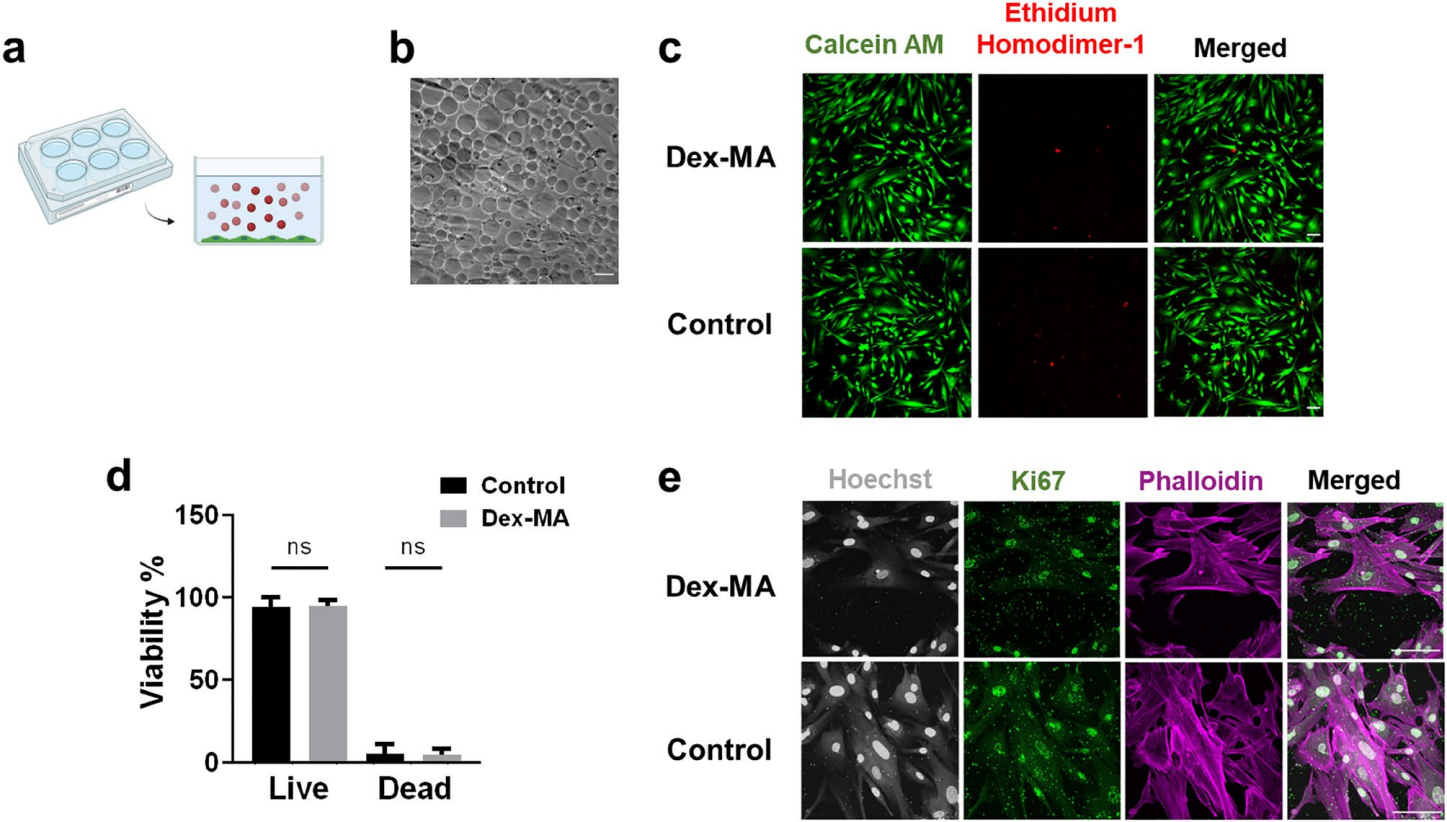
Phase separated dextran solution exhibited negligible cytotoxicity to human dermal fibroblasts. (**a**) Schematic demonstration of the setup of in vitro cell culture in the presence of phase separated Dex-MA solution, where red represents phase-separated microdomains in solution and green corresponds to attached HDFs. (**b**) Phase contrast microscope image of cells and phase separated Dex-MA (Mw: 86 kDa, f = 88%) in PBS (pH ~ 7.4) solution after seeding at 10 mg/mL concentration. (**c**) Fluorescence confocal microscope images of live/dead assay of HDF cells cultured with and without Dex-MA in DMEM media, where green color represents live cells and red color represents dead cells. (**d**) The bar plot compares cell viability in DMEM media containing Dex-MA to the control group. (**e**) Confocal microscope image of HDF cells cultured in the presence of phase-separated Dex-MA solutions in DMEM media and DMEM media without Dex-MA (control); stained with Hoechst (gray), Ki67 (green) and phalloidin (magenta), respectively. Scale bars: 100 µm.

To investigate the cyto-compatibility of Dex-MA and Dex-MA microdomains formed during the phase separation in the presence of living cells, a preliminary toxicity assay was performed. Human dermal fibroblasts (HDFs) were seeded in DMEM media with the addition of 10 mg/mL Dex-MA (86 kDa, f = 88%) (Fig. 8a) with a transition temperature of 24 °C (Fig. 2b). Incubating DMEM media containing Dex-MA at 37 °C triggered an instantaneous phase separation with microdomains dispersed in media (Fig. 8b). After 24 h of culturing, phase contrast images (Fig. 8b) revealed that HDFs were able to attach and spread on the tissue culture plastics and developed an elongated spindle morphology in the presence of phase separated Dex-MA microdomains in DMEM media. The viability of HDFs cultured in the presence of phase-separated Dex-MA solutions was compared to that of HDFs grown in DMEM media without Dex-MA. The fluorescent images in Fig. 8c showed that majority of HDFs fluoresced green with only a small fraction of cells stained red, suggesting a high cell viability (> 90%) (Fig. 8d) in both conditions. To confirm that phase-separated Dex-MA does not impact cell proliferation, we stained a proliferation marker Ki67 and demonstrated comparable numbers of proliferating HDFs cultured with or without phase-separated Dex-MA in DMEM media (Fig. 8e). Together, these data suggest that phase-separated Dex-MA microdomains do not interfere with cell attachment, spreading, or proliferation, exhibiting minimal cytotoxicity.

### Photo-crosslinking to generate microstructured hydrogels with phase-separated microdomains

**Figure 9:**
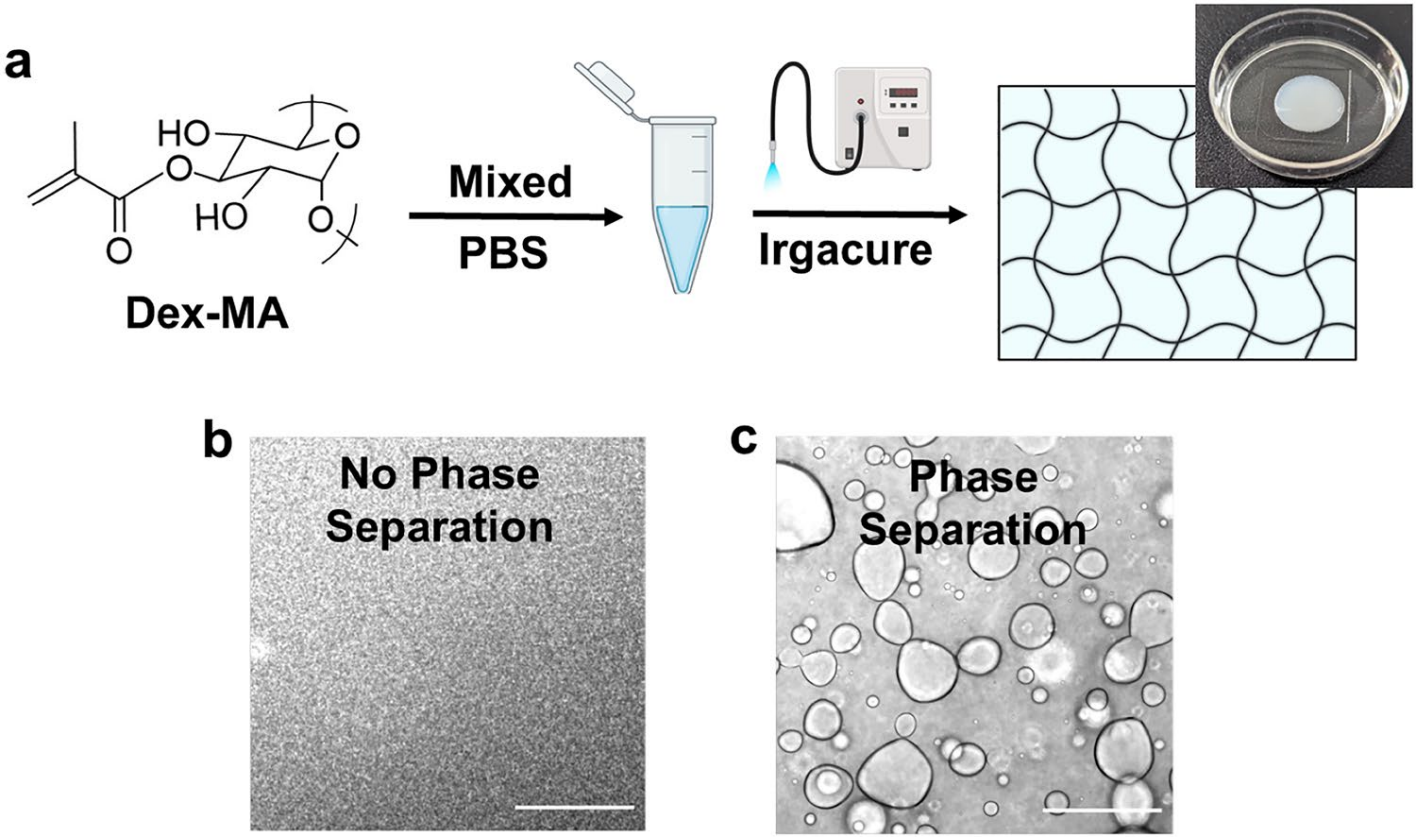
Photo crosslinking to generate microstructured hydrogels capturing phase separated microdomains. (**a**) Schematic of microstructured hydrogels formation by UV crosslinking. (**b**) Phase contrast microscope image of photo-crosslinked non-phase separated Dex-MA (Mw: 86 kDa, f = 88%) hydrogels at 50 mg/mL concentration in PBS (pH ~ 7.4) solution. (**c**) Phase contrast microscope image of photo-crosslinked phase-separated Dex-MA (Mw: 86 kDa, f = 88%) hydrogels at 50 mg/mL concentration in PBS (pH ~ 7.4) solution. Scale bar: 100 µm.

The microstructural heterogeneity of native extracellular matrix is essential in controlling the surrounding mechanical properties of cells and regulating cell behavior^53^. Incorporating such structural complexity in biomaterials with the capability to independently tune material composition would be significant^54^. To demonstrate proof-of-concept of a bottom-up approach to engineering phase separation for the formation of microstructured hydrogels, we employed a UV-initiated radical polymerization for hydrogel crosslinking (Fig. 9a). Methacrylate-facilitated photo-crosslinking chemistry permits the decoupling of phase separation kinetics from crosslinking reaction kinetics, given the rapid kinetics of crosslinking (seconds to minutes) versus that of the phase separation (minutes to hours), enabling the capture of phase-separated microdomains in bulk hydrogels. Precursor solutions of Dex-MA macromers (Mw: 86 kDa, f = 88%, 50 mg/mL in PBS) with 2% (v/v) photo-initiator (Irgacure 2959) were exposed to UV light at 365 nm wavelength for 60 s (Fig. 9a) to form elastic hydrogels, owing to the rapid reaction rates upon UV irradiation. Microstructured heterogeneous hydrogels were formed under conditions where Dex-MA precursor solutions exhibited robust LCST phase separation, and the resulting hydrogels showed distinctly dispersed phase-separated domains with micron-scale features (Fig. 9c). To generate non-phase separated hydrogels, the chemically and compositionally identical Dex-MA precursor solutions, cooled below LCST to eliminate phase separation prior to UV-crosslinking, formed homogeneous hydrogels without distinct micro-scale structures (Fig. 9b). These results demonstrate a novel and straightforward methodology to engineer phase separation in the de novo design of thermoresponsive polysaccharides for the formation of microstructured biomaterials.

## Discussion

Synthetic polysaccharides that undergo reversible phase transitions in response to temperature are of interest for developing functional thermoresponsive materials for tissue engineering and drug delivery applications^13,55^. Previous strategies to develop thermoresponsive polysaccharides relied on conjugating temperature sensitive polymers to the polysaccharides, where the selection of synthetic polymer and the degree of conjugation regulate the thermal behavior of these block-co-polymers. Although multiple parameters can tune the phase transition, it remains challenging to precisely control the transition temperature and thermal properties of these polysaccharide–polymer block-copolymers. Here, we describe a facile chemical strategy that allows gradual conjugation of hydrophobic residues to the backbone of a hydrophilic polysaccharide and generate new synthetic dextran derivatives whose macromolecular hydrophobicity can be precisely controlled to engineer phase separation. Our system addresses some of the limitations of previous material systems by eliminating the polymer block and instead leveraging chemical approaches to engineer the thermal responsiveness of conventionally non-temperature sensitive polysaccharides. A recent study also modified the dextran backbone with alkylamide derivatives and leveraged thermally reversible H-bonding from alkylamide groups with water molecules to induce thermoresponsive properties, a similar approach to ours. However, their chemical process necessitates two stages of chemical conjugations, whereas our system requires only a single, simplified step of chemical modification to engineer hydrophobicity, thereby inducing a robust and comparable LCST^13^. Native dextran does not exhibit temperature-dependent phase separation due to ample hydroxyl groups on the polymer backbone, however, attaching hydrophobic methacrylate groups to the dextran backbone through these hydroxyl residues increases the overall macromolecular hydrophobicity that triggers reversible phase separation in aqueous solution. By systematically increasing the level of methacrylation functionality from 40 to 88%, we reveal that the extent of macromolecular hydrophobicity directly controls de novo designed phase separation where a critical degree of methacrylation 70% is required in order to induce effective and reversible phase transition.

Phase separation, particularly temperature induced, has previously been suggested to be an entropy driven process in that interactions between polymers and water molecules are favorable through robust hydrogen bonding at low temperature, whereas at high temperature, polymer-water interactions became energetically unfavorable and this disruption of the hydrogen bonding favors intermolecular interactions between hydrophobic entities resulting in solute dehydration, insolubility and ultimately aggregation^28,56,57^. The minimum 70% of methacrylate functionality needed to drive the phase separation suggests that methacrylation percentage below 70% does not exert sufficient hydrophobicity to disrupt the water molecules around dextran backbone at higher temperatures to demonstrate effective phase transition (Fig. 2b) likely due to dextran’s intrinsic hydrophilicity. Notably, the transition temperatures of these engineered thermoresponsive dextrans can be further modulated by various material properties and solution conditions e.g., concentration, molecular weight, degree of methacrylation (Figs. 2 and 3). For example, the concentration dependent LCST behavior of methacrylated dextrans is in general consistent with other thermoresponsive materials such as synthetic polymers (e.g. poly(N,N-diethylacrylamide) (PDEAM), Poly(methyl vinyl ether) (PMVE), Poly-N-vinylcaprolactam (PNVCL)), native polysaccharides (e.g., methylcellulose, chitosan, xyloglucan), recombinant elastomeric structural polypeptides (e.g., ELPs, RLPs and SLPs) and dextran-based copolymers (e.g., dextran grafted poly(N-vinylcaprolactam) block copolymers)^1,13,40,41,46,58–66^, where the transition temperature decreases with the increase of polymer concentration^41,67^. The fast reversible phase transition behavior of Dex-MA solution with minimal hysteresis indicates the rehydration and dissolution of microdomains, making the system a potentially useful thermoresponsive material.

Numerous studies have suggested that thermoresponsive behavior is influenced by the hydrophobic-hydrophilic balance within macromolecules and stability of hydration between macromolecules and water molecules, and the addition of surfactant, urea and salts could change the phase transition by altering that balance^57^. Given both hydrogen bonding and hydrophobic-hydrophobic interactions are involved during phase separation in our system, we employed urea and SDS to modulate the phase transition behavior and observed both materials indeed regulating the coalescence of particles to form aggregates at transition temperatures (Fig. 4a–c), however, while 0.01 M SDS can effectively eliminate phase transition, a minimum of 4 M urea was required to abolish Dex-MA phase separation. This observation of very high sensitivity to SDS compared to urea supported our hypothesis that enhanced hydrophobic-hydrophobic interaction is the dominant driving force to trigger phase separation in our system.

Hofmeister series have also been extensively studied to alter the transition temperature of various materials including proteins and polypeptides^41,68^. Hofmeister effect defines the order of Chaotropic ions (salting-in) and Kosmotropic ions (salting-out) based on the ion’s hydration strength in stabilizing or destabilizing polymer solubility during phase separation^51^. By comparing the thermal behavior of Dex-MA aqueous solutions across both series of Hofmeister salts, we observed that all cations characterized had a negligible impact on the phase transition temperatures of Dex-MA solutions while anions induced significant effects on the transition temperatures (Fig. 5). These findings suggest that anions appear to have a profound effect on the LCST behavior of these materials compared to cations, which has been previously observed in many other systems^26,52^. The discrepancy between Hofmeister anions and cations is in part due to the large size, high polarization capability and different hydration characteristics^52^. Our data is also consistent with the order of Hofmeister anionic series SO_4_^2^ > S_2_O_3_^−^ > HPO_4_^2^ > Cl^−^ > Br^−^ > NO_3_^−^ > I^−^ > SCN^−^; where Kosmotropic anions demonstrated a salting out effect strengthening hydrophobic-hydrophobic interactions that correlates with a lower transition temperature of Dex-MA whilst Chaotropic anions yielded a salting in effect increasing the solubility of nonpolar molecules that results in increased transition temperatures. The pattern in the Hofmeister salt series shown in dextran-MA is consistent to those seen in other polymers such as various ELPs and pNIPAM^26,68^. We do, however, observe that the addition of NaCl salts at various ionic strengths significantly lowered the transition temperature, which is in stark contrast to what has been observed in RLPs and is likely caused by ion pairing affecting both electrostatic and hydrophobic interaction^41^.

A material system where phase transition can be systematically tuned and defined without multiple steps of chemical conjugation or purification would have broad utility. Modulating hydrophobicity has been classically used to tune the transition temperatures of recombinant proteins through introducing hydrophobic amino acid guest residues. Although this strategy has been successful, each modification requires genetic engineering and recombinant synthesis of a new construct that is time consuming. It is also challenging to precisely control the phase transition due to the complexity in chemical compositions and conformational changes in the resulting proteins. Our method to modify macromolecular hydrophobicity by introducing hydrophobic adducts to hydrophilic polysaccharide dextran backbones not only effectively induces reversible phase transition, but also easily tunes the transition temperatures and phase-separated microdomain sizes by defined chemical modification. The precise control of methacrylation functionality permits the tuning of macromolecular hydrophobicity and independent manipulation of transition temperatures in a well-defined manner, making it advantageous compared to traditional co-polymer grafting approaches in many other systems. In addition, we also demonstrated the feasibility and applicability of leveraging this simple one step chemical modification to trigger phase separation and form phase-separated microstructured hydrogels that avoids the need of any specific instrument (e.g., microfluidics, photolithography, or batch emulsion). Future work will systematically investigate the phase transition process, delineate the boundary conditions and fabricate heterogeneous hydrogels with tunable microstructures to enhance cell–matrix interactions. Stemming from the results of this present work, the versatility of our approach can be generalized and can essentially convert any non-thermoresponsive polysaccharide to become thermoresponsive, and further these engineered polysaccharides offer compelling alternatives in diverse fields of biomedical engineering.

## Conclusion

In this work, we describe a facile strategy to engineer macromolecular hydrophobicity to induce reversible phase transition in hydrophilic dextran through chemical modification of the polysaccharide with hydrophobic residues. Systematic substitution of hydroxyl groups with methacrylate residues on dextran backbone gradually increases the overall hydrophobicity of modified macromers. The reversible phase separation of methacrylated dextran solution features an LCST which can be tuned via multiple variables including temperature, concentration, molecular weight, degree of methacrylation, Hofmeister salt identity, ionic strength, surfactant and urea. Morphological characterization of phase separated Dex-MA solutions revealed the formation of microdomains whose size can also be modulated via initial solution conditions. The phase-separating dextran exhibited minimal cytotoxicity and had minimal impact on cell viability of cultured human dermal fibroblast cells. Microstructured hydrogels can be formed by locking the phase-separated microdomains via UV crosslinking. The strategy of introducing hydrophobic moieties to polymer backbones to convert conventional hydrophilic macromolecules to hydrophobic counterparts offers a new route to generate thermoresponsive polysaccharides, an approach that is drastically different from traditional polymer-grafting polysaccharide copolymers. These advanced temperature-sensitive polysaccharides offer new opportunities to generate stimuli-responsive, heterogeneous microstructured biomaterials in tissue engineering.

### Supplementary Information

Supplementary Figures.

## Data Availability

The datasets used and/or analyzed during the current study are available from the corresponding author on reasonable request.
