# Supplementary material for: Inducing an LCST in hydrophilic polysaccharides via engineered macromolecular hydrophobicity
Source: Sci Rep. 2023 Sep 9;13:14896. doi: 10.1038/s41598-023-41947-z (PMC10492858; doi:10.1038/s41598-023-41947-z)
Supplement: Supplementary file 1 — Supplementary Figures. [file 41598_2023_41947_MOESM1_ESM.docx]

**Supporting Information**

**Inducing an LCST in Hydrophilic Polysaccharides *via* Engineered Macromolecular Hydrophobicity**

Saniya Yesmin Bubli^1^, Matthew Smolag^1^, Ellen Blackwell^1^, Yung-Chun Lin^2^, John G. Tsavalas^2, 3^ and Linqing Li^1,^ *

^1^Department of Chemical Engineering and Bioengineering, University of New Hampshire, Durham, NH 03824, USA; ^2^Department of Chemistry, University of New Hampshire, Durham, NH 03824, USA; ^3^Materials Science Program, University of New Hampshire, Durham, NH 03824, USA

*Corresponding author: Linqing.Li@unh.edu


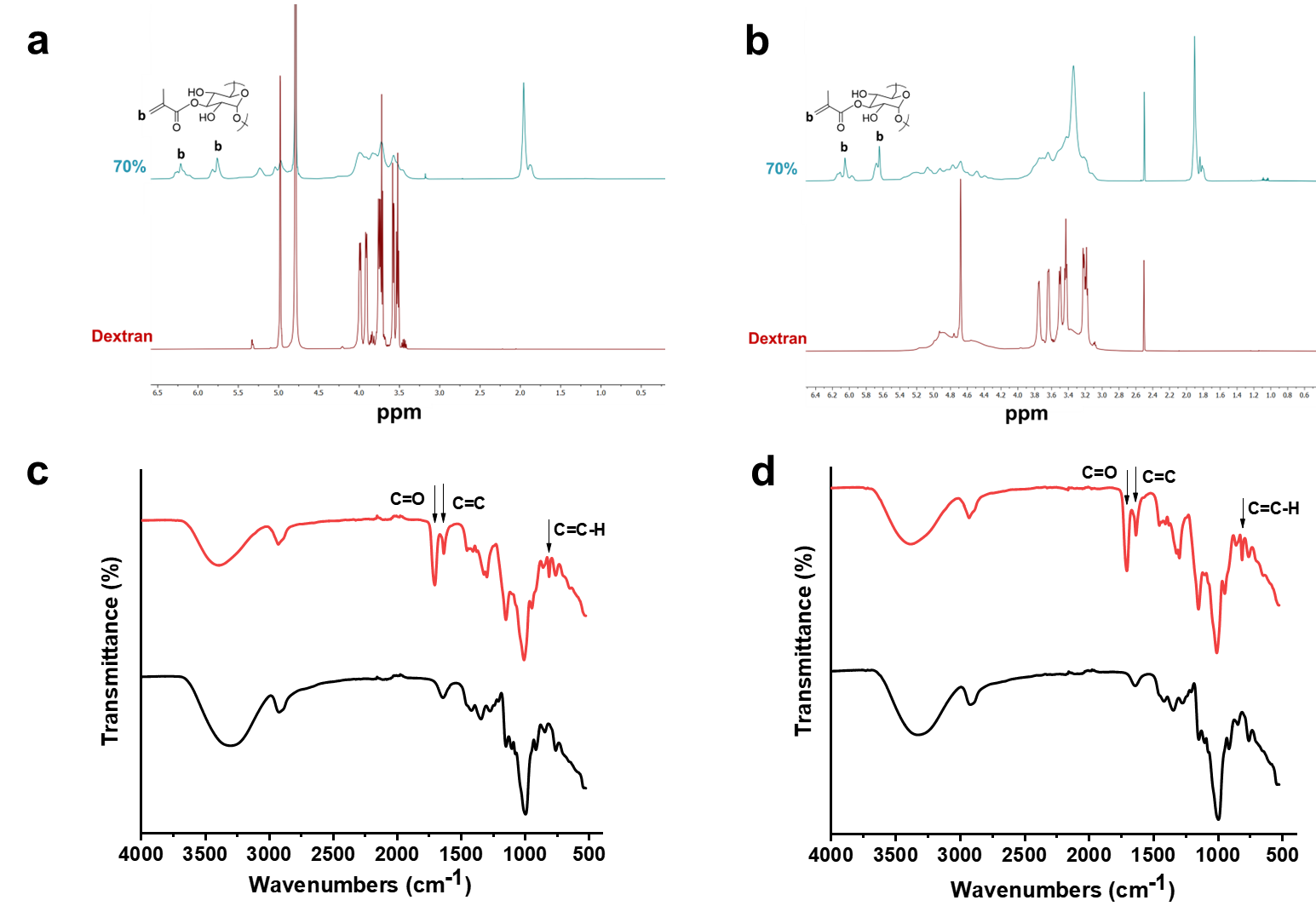


**Figure S1. Characterization of dextran methacrylate samples.** H-NMR spectrum of dextran and Dex-MA with 70% methacrylation for molecular weights **(a)** 250kDa (in D_2_O) and **(b)** 500 kDa (in DMSO). FTIR spectrum of dextran and Dex-MA with 70% degree of methacrylation for molecular weights **(c)** 250kDa and **(d)** 500 kDa.


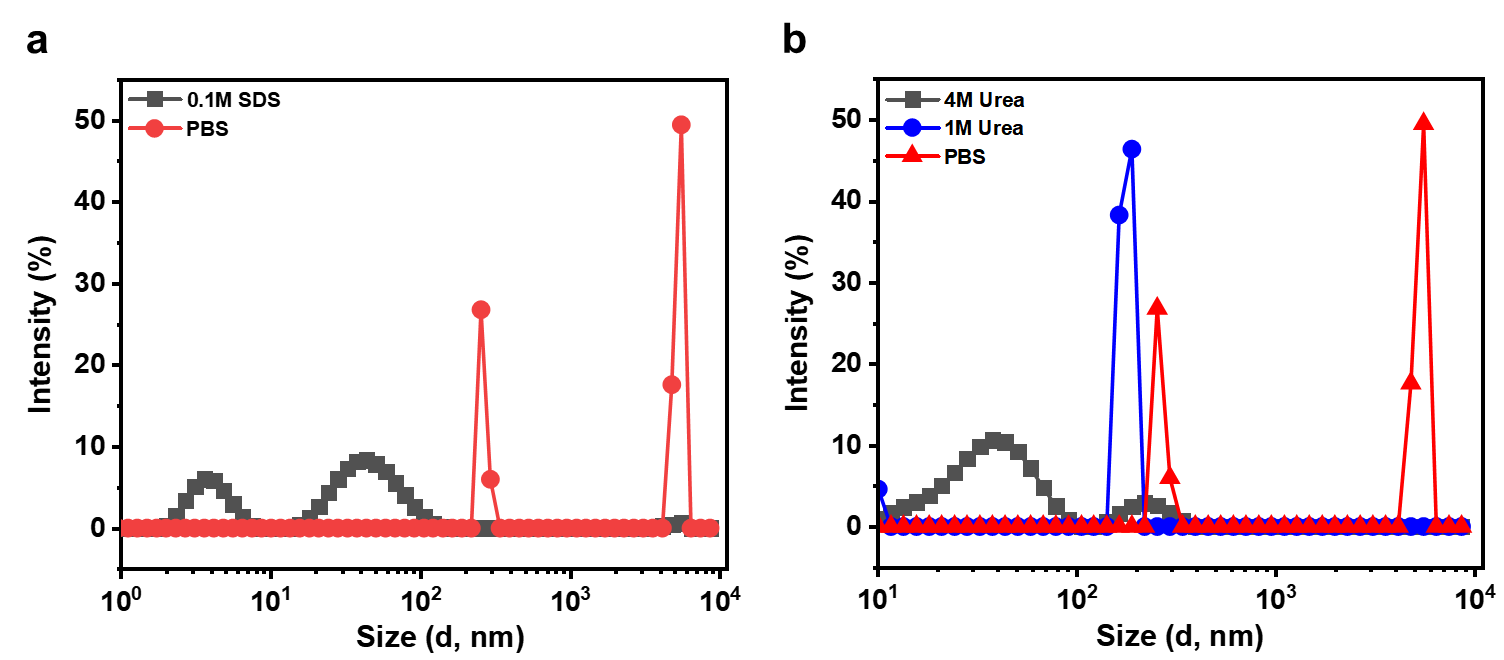


**Figure S2.** **Dynamic light scattering characterization of Dex-MA phase transition in aqueous solutions of PBS (pH~7.4), surfactant and urea. (a)** Intensity (%) of Dex-MA solutions at 1 mg/mL concentration in SDS (0.1M) and PBS as a function of size (Mw: 86 kDa, f = 88%). **(b)** Intensity (%) of Dex-MA solutions at 1 mg/mL concentration in urea (4M and 1M) and PBS as a function of size (Mw: 86 kDa, f = 88%).

**a. 0.1 mg/mL**

**
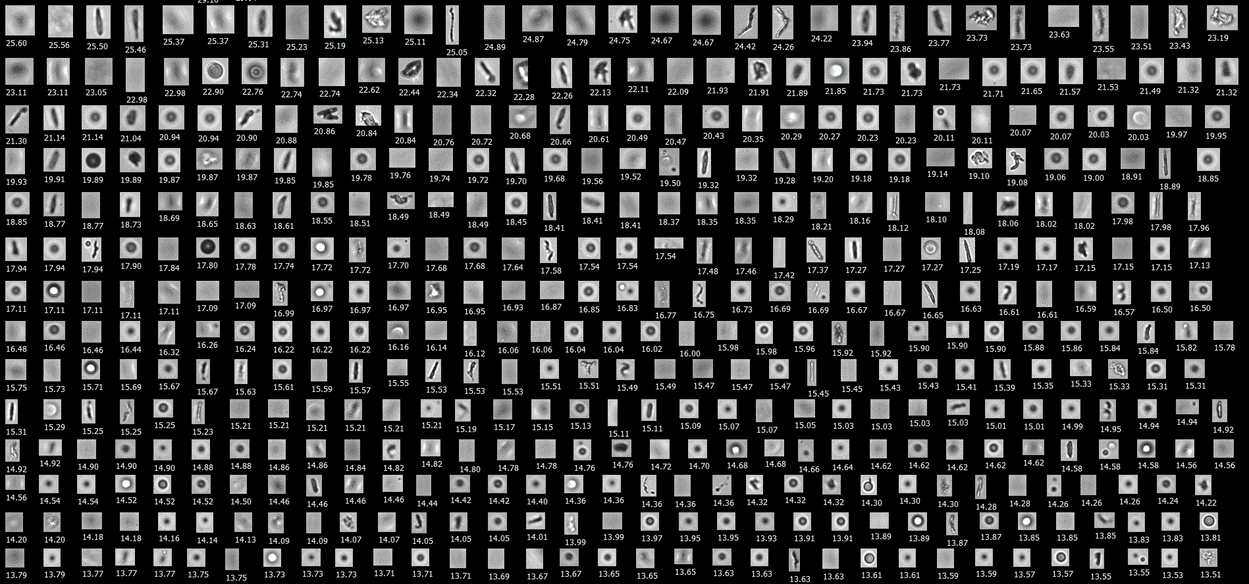
**

**b. 1 mg/mL**


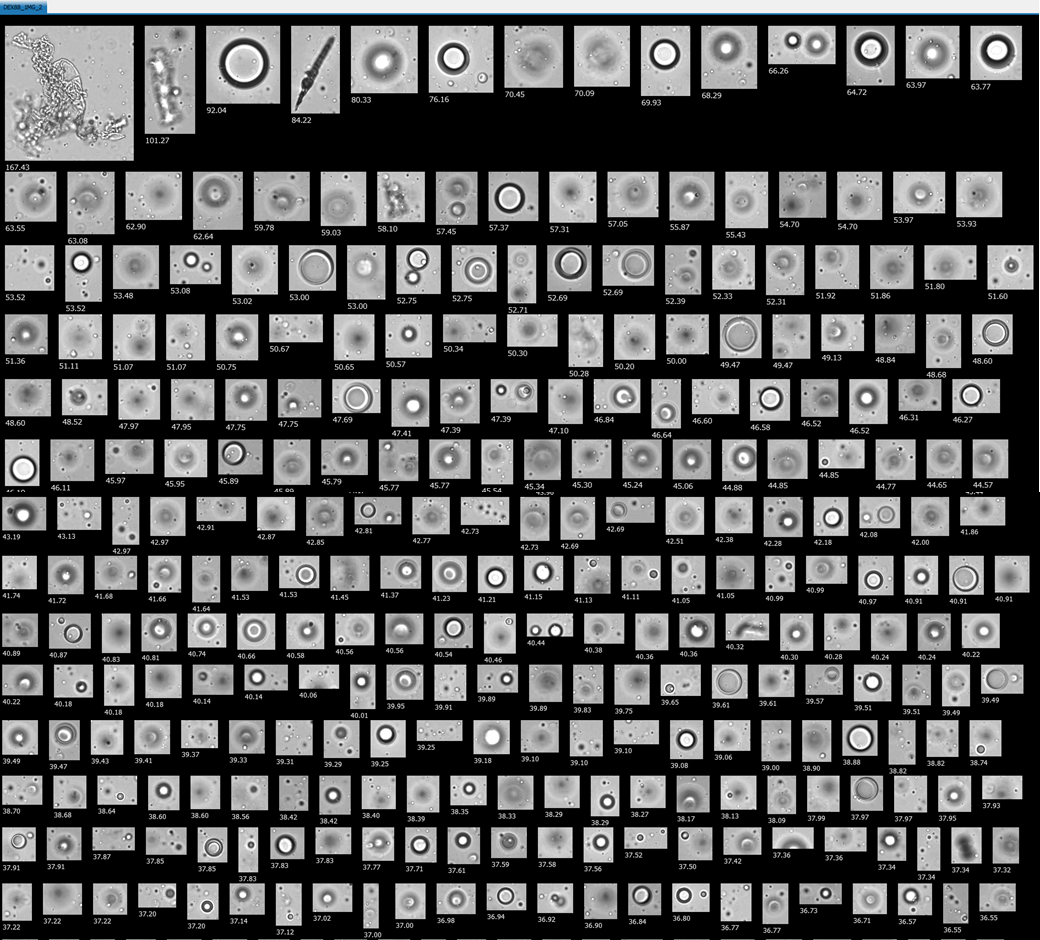


**c. 10 mg/mL**


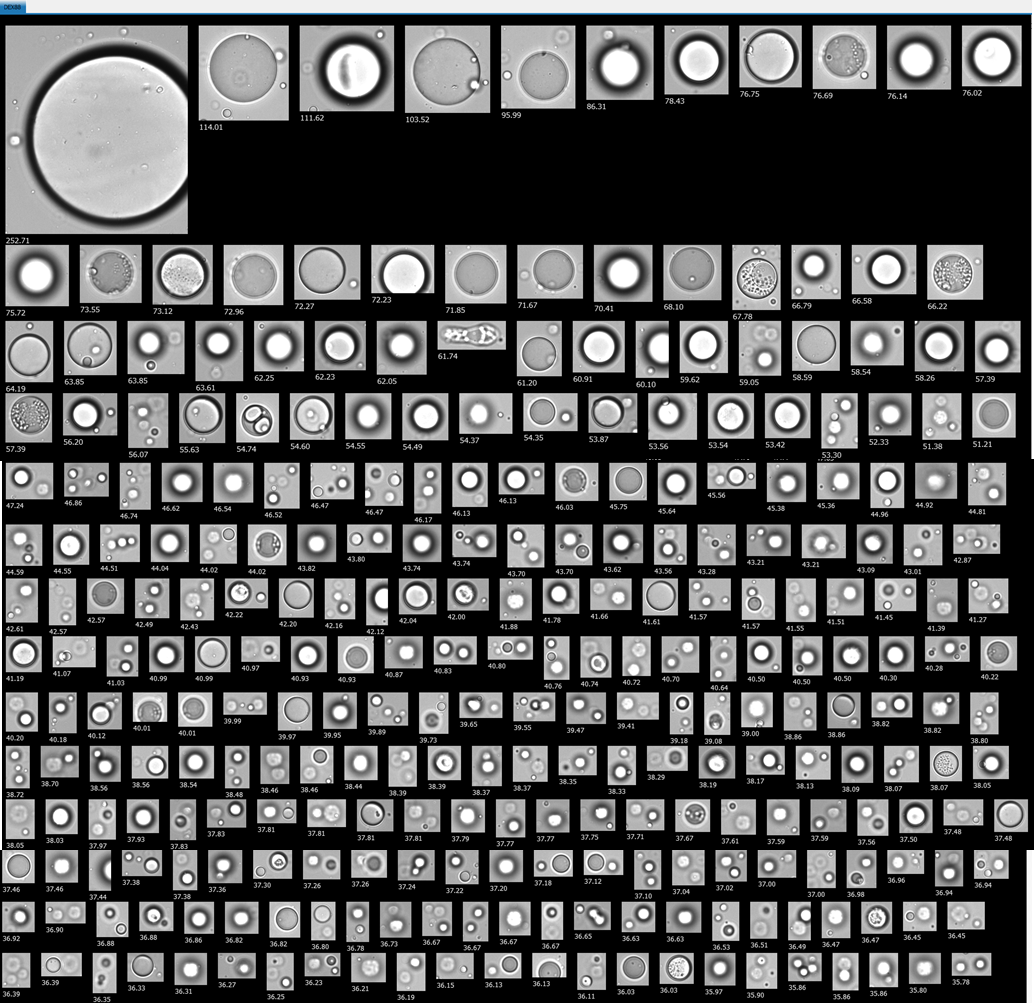


**Figure S3.** **Raw images of phase-separated microdomains in aqueous Dex-MA solution *via* flow imaging microscopy**. Representative FIM images of Dex-MA in PBS (pH~7.4) solutions (Mw: 86kDa, f = 88%) at **(a)** 0.1 mg/mL, **(b)** 1 mg/mL and **(c)** 10 mg/mL concentrations.
